# DCE-MRI radiomics nomogram can predict response to neoadjuvant chemotherapy in esophageal cancer

**DOI:** 10.1007/s12672-022-00464-7

**Published:** 2022-01-08

**Authors:** Jinrong Qu, Ling Ma, Yanan Lu, Zhaoqi Wang, Jia Guo, Hongkai Zhang, Xu Yan, Hui Liu, Ihab R. Kamel, Jianjun Qin, Hailiang Li

**Affiliations:** 1 Department of Radiology, Affiliated Cancer Hospital of Zhengzhou University and Henan Cancer Hospital, Zhengzhou, 450008 Henan China; 2 Advanced Application Team, GE Healthcare, Shanghai, 201203 China; 3 Department of Thoracic Surgery, Affiliated Cancer Hospital of Zhengzhou University, Zhengzhou, 450008 Henan China; 4 Present Address: Department of Thoracic Surgery, National Cancer Center/Cancer Hospital, Chinese Academy of Medical Sciences and Peking Union Medical College, Beijing, 100021 China; 5 NEA MR Collaboration, Siemens Ltd., China, Shanghai, 201318 China; 6 Department of Radiology, Johns Hopkins University School of Medicine, Baltimore, MD 21205-2196 USA

**Keywords:** Esophageal cancer, Magnetic Resonance Imaging, Adjuvant chemotherapy, Nomograms, Precision medicine

## Abstract

**Objectives:**

To assess volumetric DCE-MRI radiomics nomogram in predicting response to neoadjuvant chemotherapy (nCT) in EC patients.

**Methods:**

This retrospective analysis of a prospective study enrolled EC patients with stage cT1N + M0 or cT2-4aN0-3M0 who received DCE-MRI within 7 days before chemotherapy, followed by surgery. Response assessment was graded from 1 to 5 according to the tumor regression grade (TRG). Patients were stratified into responders (TRG1 + 2) and non-responders (TRG3 + 4 + 5). 72 radiomics features and vascular permeability parameters were extracted from DCE-MRI. The discriminating performance was assessed with ROC. Decision curve analysis (DCA) was used for comparing three different models.

**Results:**

This cohort included 82 patients, and 72 tumor radiomics features and vascular permeability parameters acquired from DCE-MRI. mRMR and LASSO were performed to choose the optimized subset of radiomics features, and 3 features were selected to create the radiomics signature that were significantly associated with response (*P* < 0.001). AUC of combining radiomics signature and DCE-MRI performance in the training (n = 41) and validation (n = 41) cohort was 0.84 (95% CI 0.57–1) and 0.86 (95% CI 0.74–0.97), respectively. This combined model showed the best discrimination between responders and non-responders, and showed the highest positive and positive predictive value in both training set and test set.

**Conclusions:**

The radiomics features are useful for nCT response prediction in EC patients.

## Introduction

Esophageal cancer (EC) is the sixth leading cause of cancer mortality globally [1]. Neoadjuvant therapy combined with surgery has become the standard treatment for local advanced EC [2, 3], and which includes neoadjuvant chemoradiotherapy (nCRT) and neoadjuvant chemotherapy (nCT) [4, 5]. Although it has been reported that nCRT could achieve more pathologic complete response (pCR) than nCT, an updated meta-analysis showed no clear advantage of nCRT over nCT [6]. In western countries, ECs are mainly adenocarcinoma, and nCRT is the treatment of choice [7]. However, in China and Japan, almost all the ECs are squamous cell carcinoma, and nCT is the treatment of choice particularly for stages II and III [5]. Although nCT could improve overall survival, surgery is still important especially for non-responsive patients [8]. However, pretreatment prediction of response to nCT in EC remains challenging.

Radiomics could provide more information than conventional images, and pretreatment 18F-FDG PET and MRI radiomics have been used for predicting outcome of patients with locally advanced cervical cancer treated with chemoradiotherapy [9], as well as in rectal cancer [10]. Meanwhile, MRI radiomics showed individualized estimation of lymph node metastasis in EC patients [11]. Dynamic contrast enhanced (DCE)-MRI represents tumor perfusion by contrast media, and it has an encouraging role in predicting tumor response to nCRT and patient survival [4, 12]. Recent improvements in DCE-MRI allow for high quality imaging of the chest during free-breathing. However, to date there is no report about volumetric DCE-MRI radiomics nomogram that could predict response of EC patients to nCT. Therefore, our goal was to develop and validate such a volumetric DCE-MRI signature of primary tumor and determine if this signature could predict tumor histopathologic response in patients with EC treated with nCT.

## Methods

### Patients

Pretreatment staging was evaluated with measures including physical examination, standard laboratory tests, pulmonary function tests, esophagogastroduodenoscopy (EGD) with endoscopic ultrasound and biopsy, chest/abdominal CT with contrast, and PET if available. A multidisciplinary team was employed for patients’ evaluation before treatment, as stated by institutional practice guidelines between September 2016 and March 2018. Patients had histologically proven and resectable EC with stage cT1N + M0 or cT2-4aN0-3M0, according to the 7th TNM staging system of the American Joint Committee on Cancer [13]. The consecutive patients were retrospectively evaluated, and this analysis of a prospective study (ChiCTR-DOD-14005308) was approved by the institutional review board and informed consent was performed by all patients.

### MRI technique

All patients received DCE-MR before chemotherapy, which was performed within 1 week before the beginning of chemotherapy, and the time interval between initial staging and pretreatment MRI was 1–6 days.

### Positioning coil selection and examination

MRI examination in a 3 T MR scanner (MAGNETOM Skyra, Siemens Healthcare, Erlangen, Germany) with DCE radial VIBE with 3 mm slice thickness. A prototype dynamic radial VIBE sequence with a total of 68 periods and 4896 images were collected, as every period included 72 images, was performed for the whole chest during free breathing with the total scanning time of 309 s. At 20 s after the beginning of scanning, 10–15 mL Gadopentetate Dimeglumine Injection (0.2 ml/kg of body weight, Omniscan, GE Healthcare) was injected at a rate of 2.5 mL/s by a MR-compatible automated double-tube high-pressure injector (Spectris Solaris EP, Medrad, Indianola, PA), and equal volume of normal saline solution was used to flush the tube.

### Chemotherapy protocol

All patients received 2 cycles of standard neoadjuvant paclitaxel and nedaplatin protocol followed by surgical resection, as published in a prior study [14]. The median time from the completion of chemotherapy to surgery was 23 days (range 21–30 days).

### Pathology

Tumor regression grade (TRG) as described by the Mandard classification [15] was used to classify chemotherapy response assessment into 5 stages, including: TRG1 (no residual cancer cells), TRG2 (rare residual cancer cells), and TRG3 (fibrosis outgrowing residual cancer), TRG4 (residual cancer outgrowing fibrosis), and TRG5 (absence of regressive changes). A total of 82 patients had TRG available for this study and they were stratified into a responsive group (TRG1 + 2) and a non-responsive group (TRG3 + 4 + 5).

### Radiomics workflow

**Fig. 1:**
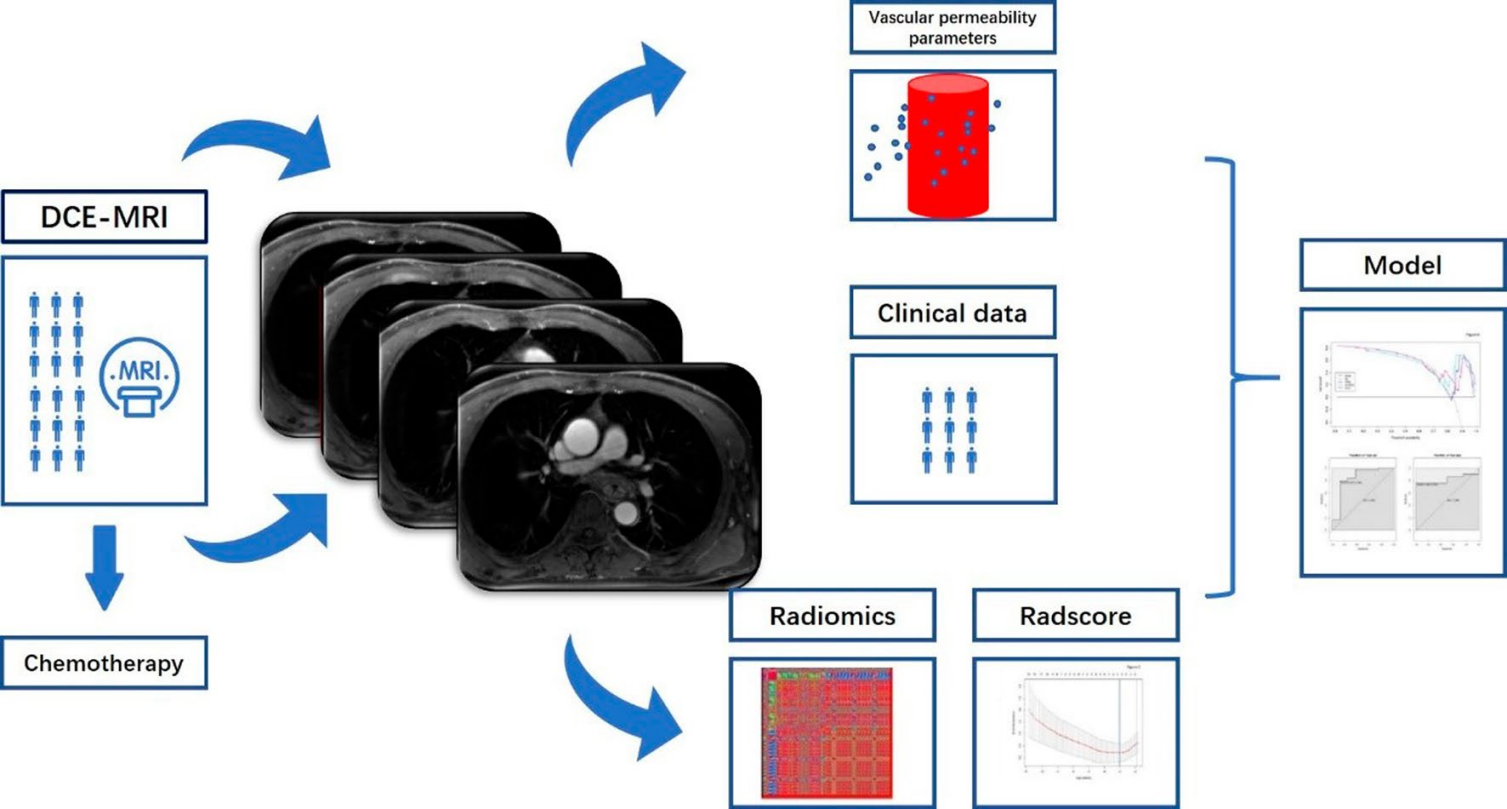
Radiomics workflow

Radiomics workflow of this study is presented in Fig. 1, including the following procedures: (1) DCE-MRI scanning, (2) chemotherapy, (3) DCE-MRI scanning, (4) vascular permeability parameters extraction, (5) radiomics feature extraction, (6) Radscore building, (7) building model and validating model.

### Tumor segmentation

MR signal is usually relative, with large differences between scanners and vendors. By normalizing the image before feature calculation, this confounding effect may be reduced. The equation of image standardization as follow:

$${\text{Image standardization}} = \frac{x - \mu }{{adjusted\,stddec}}$$

$$adjusted\,stddev = {\text{max}}\left( {\sigma ,\frac{1.0}{{\surd N}}} \right)$$

μ is the average level of signal for images. X is the matrix of images. All images have been resampled to 1 × 1 × 1 mm^3^. DCE-MRI features analysis was performed on motion corrected data with an in-house developed software named Omni-kinetics (GE Healthcare, China). Arterial input function (AIF) was obtained by placing a regions of interest (ROI) on the thoracic aorta in the peak arterial enhancement phase. An Extended Tofts Linear model was used to generate the permeability parameters. 3D tumors of pre-nCT were segmented by two radiologists (with 15 and 12 years of experience in MR imaging) by consensus (Appendix Fig. 6), who were blinded to the pathology results. The reviewers carefully segmented the entire tumor by manually contouring the whole tumor on each tumor slice. All large vessels and/or necrotic area(s) were avoided. The resulting 3D ROIs were used for subsequent extraction of radiomic features, and vascular permeability parameters were recorded.

### Radiomics feature extraction

Radiomics features were computed based on the 3D ROIs segmentation results using a locally developed scientific software Omni-kinetics (GE Healthcare, Shanghai, China) that is not commercially available. Features were categorized into five primary subgroups: (1) 14 first-order, (2) 13 histogram, (3) 13 GLCM, (4) 16 RLM, and (5) 16 pharmacokinetic parameters. Intra-reader agreement of each radiomics feature was assessed by Inter-class correlation coefficient (ICC), and ICC with greater than 0.70 was considered a good agreement.

### Statistics analysis

This group of research uses R (version 3.8.1) for processing and analysis, and uses “xml2” (read data), “tidyverse” (data visualization, data cleaning), “caret” (data preprocessing, feature selection), and “pROC” (model evaluation)), “glmnet” (LASSO, Logistic model), “DMwR” (uneven sample classification), “rmda” (clinical decision curve), “ggpubr” (data grouping), “ModelGood” (model evaluation), “rms” (Nomogram drawing), “mRMRe” (mRMR), “DescTools” (descriptive statistical analysis), “Publish” (logistic regression result) data package. Kologoroy Smirnov is used to test whether the measurement data conform to the normal distribution. The measurement data conforming to the normal distribution are represented by the mean ± standard deviation, and the measurement data not conforming to the normal distribution are represented by the median. Enumeration data were compared by X2 test, and measurement data were compared by independent sample t test or Mann–Whitney U test. P < 0.05, the difference is statistically significant. The effectiveness of the scoring system was evaluated based on the area under the ROC curve AUC.

### Dataset split and demographic comparison

According to the ratio of 5:5, one half of this cohort (41 patients including 35 non-responders and 6 responders) was set as the training cohort, and the other half (41 patients including 35 non-responders and 6 responders) was set as the test cohort. We used independent samples t-test and Mann–Whitney U test to evaluate the differences in age between the training set and test set, while chi-square tests were used to compare the differences in TRG, clinical characteristics and combined clinical characteristics and TRG (gender, clinical T-stage, N-stage, type, tumor location, and TRG).

### Features redundancy and radiomic signature building

Due to the high dimensionality of these imaging texture features, we only used stable features with ICCs value ≥ 0.70 since these variables had good reproducibility.

The selected texture features were identified by one-way analysis of variance, Mann–Whitney U test for classifying responders and non-responders in the training set. We used two feature selection method, mRMR and LASSO to select the feature. At first, mRMR was performed to eliminate the redundant and irrelevant features, and 30 features were retained. Then LASSO was conducted to choose the optimized subset of features to construct the final model. In order to assess the predicting performance of radiomics for each patient, a radiomics score (referred to as Rad-score) was computed for each patient, and the weight of each feature is determined by LASSO logistic with tenfold cross-validation which were obtained from the training set. The difference of Rad-score between the training and test set was analyzed using Mann–Whitney U (non-parametric, unpaired) test. We also performed univariable association analysis between the Rad-score and response in both training and test sets.

### Radiomics nomogram development

Multimodal logistic regression analysis started with a responsive group and a non-responsive group, clinical characteristics with the following candidate variables: gender, age, clinical T-stage, clinical N-stage, type and tumor location, and combined clinical characteristics and TRG. In order to provide a clinically useful tool which is able to predict the pathologic response to nCT probability of each patient, three nomograms of radiomics, DCE-MRI, and combined radiomics and DCE-MRI were generated based on multivariate analysis in the training set. All the features of DCE-MRI would be included in the multimodal logistic regression model based on the variance inflation factor (VIF) that the threshold value of VIF is 10.

### Evaluation of prediction performance of the nomogram

The performance of the resulting radiomics nomogram were first evaluated using area under curve (AUC) in the training set and then validated in the test set. The Mann–Whitney U test was adopted for testing the potential correlation of the radiomics signature and response in the training cohort, and decision curve analysis (DCA) was used for comparing three models that were built with Radscore, DCE-MRI and the combination of Radscore and DCE-MRI. The positive predictive value (PPV) and negative predictive value (NPV) of different models were calculated.

## Results

### Patient characteristics

Patient characteristics in the training set and test set are shown in Table 1, and responders both in the training set and test set was 14.6% (6/41). According to independent samples t-test, there were no significant differences in demographic characteristics between the training set and test set.

**Table 1:** Patient and treatment-related characteristics with pre-nCT EC in the training set and test set (n = 82)

	Training set		Test set		P
	responders	non-responders	responders	non-responders	
Gender					0.563
Male	3	32	5	18	
Female	3	3	1	17	
Age, years	56.8 ± 9.4	59.4 ± 7.9	62.0 ± 7.6	59.8 ± 7.9	0.545
Clinical T-stage					0.198
T1	0	0	1	0	
T2	1	7	3	8	
T3	5	23	2	24	
T4	0	5	0	3	
Clinical N-stage					0.429
No	5	18	4	14	
N1	1	6	1	11	
N2	0	10	1	7	
N3	0	1	0	3	
Type					0.588
SCC	6	33	6	32	
AC	0	1	0	1	
ASC	0	1	0	2	
Location					0.712
Upper third of esophagus	1	6	2	5	
Middle third of esophagus	4	21	2	24	
Distal third of esophagus	1	8	2	6	
TRG					1.000
TRG 1	3	0	1	0	
TRG 2	3	0	5	0	
TRG 3	0	3	0	2	
TRG 4	0	5	0	9	
TRG 5	0	27	0	24	
Tumor size					
Max size(cm)	0.428 ± 0.210	0.222 ± 0.239	0.178 ± 0.194	0.132 ± 0.143	0.376

SCC, squamous cell carcinoma; AC, adenocarcinoma; ASC, adenosquamous carcinoma

### Selection of candidate radiomics features and building a radiomics signature

**Fig. 2:**
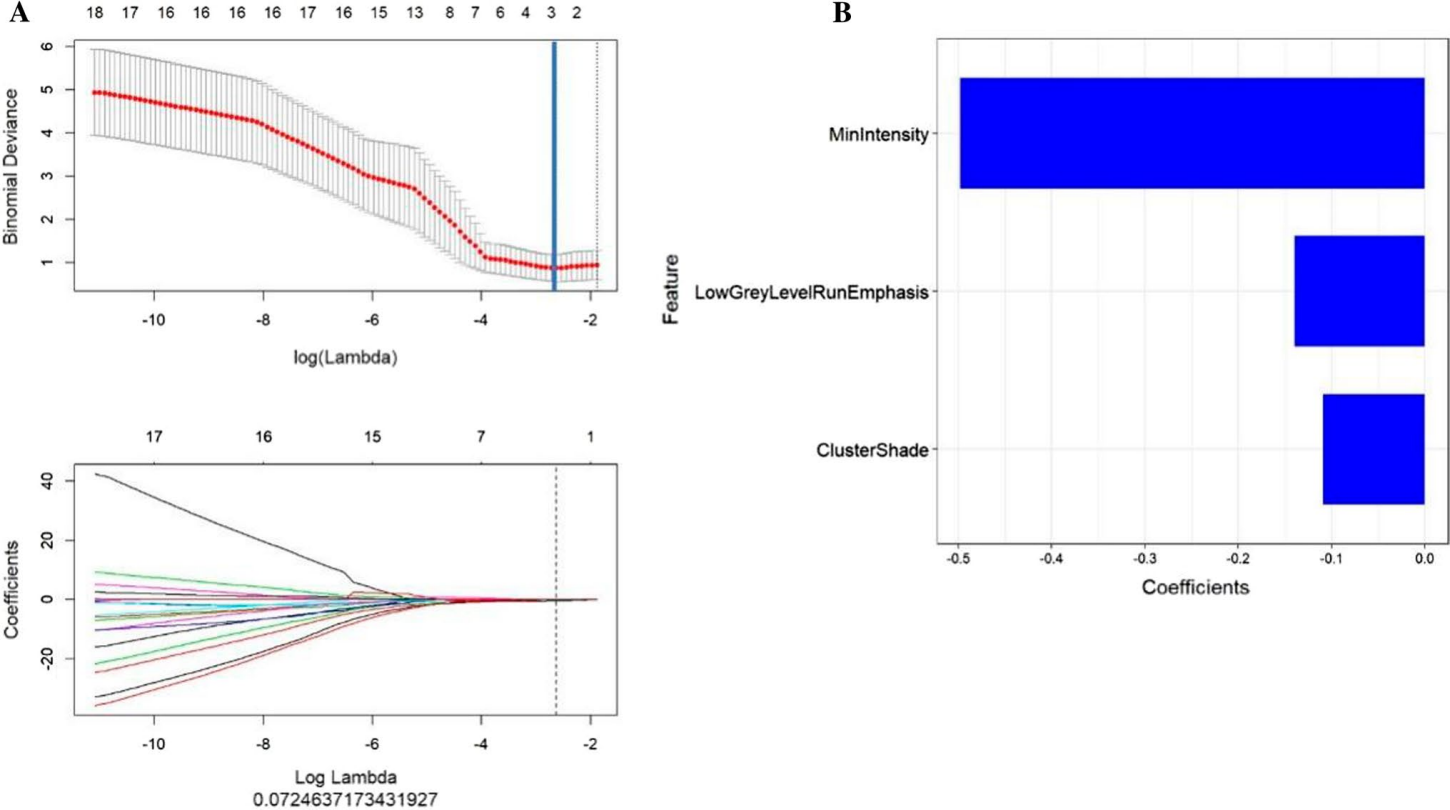
**A** selection of response-associated radiomics features via LASSO algorithm, and showing the cross-validation curve. Blue vertical lines were drawn at the optimal value by using tenfold cross-validation and the 1 standard error of the minimum criteria (the 1-SE criteria). An optimal lambda value of 1.181, with log (lambda) = 0.0724, was selected, and 3 nonzero coefficients were chosen. **B** the most predictive subset of radiomics features for predicting response to nCT

**Fig. 3:**
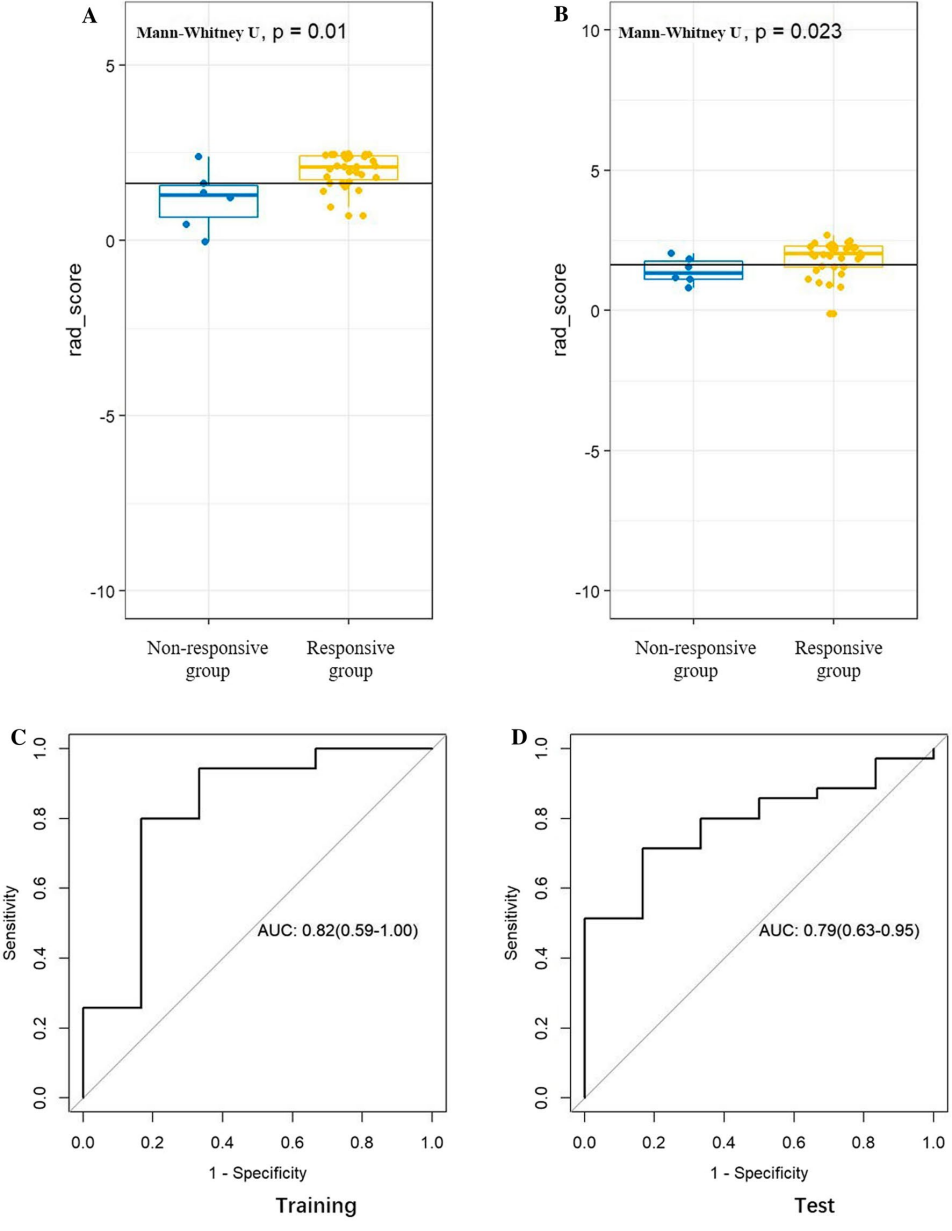
The radiomics score between responsive group and non-responsive group for each patient in the training set (**A**) and test set (**B**). ROCs of radiomics model for predicting response to nCT on training set (**C**) and test set (**D**) respectively

After feature dimension reduction, 3 features were used to construct Rad-score which is a radiomics signature [16]. These 3 features were MinIntensity, LowGreyLevelRunEmphasis, ClusterShade, and were selected for classifying responders and non-responders in the training set (Fig. 2). The Rad-score was calculated according to the following formula: (Fig. 3)

$$Rad - score = 0.498*MinIntensity + - 0.11*ClusterShade + - 0.14*LowGreyLevelRunEmphasis + 1.887$$

The radiomics signatures showed the significant difference between responsive group and non-responsive group in the Training set (p = 0.01, Fig. 3A) and Testing set (0.023, Fig. 3B) with the AUC is 0.82 and 0.79 in Training set (Fig. 3C) and Testing set (Fig. 3D).

### Development, validation, and performance of a predictive nomogram

**Fig. 4:**
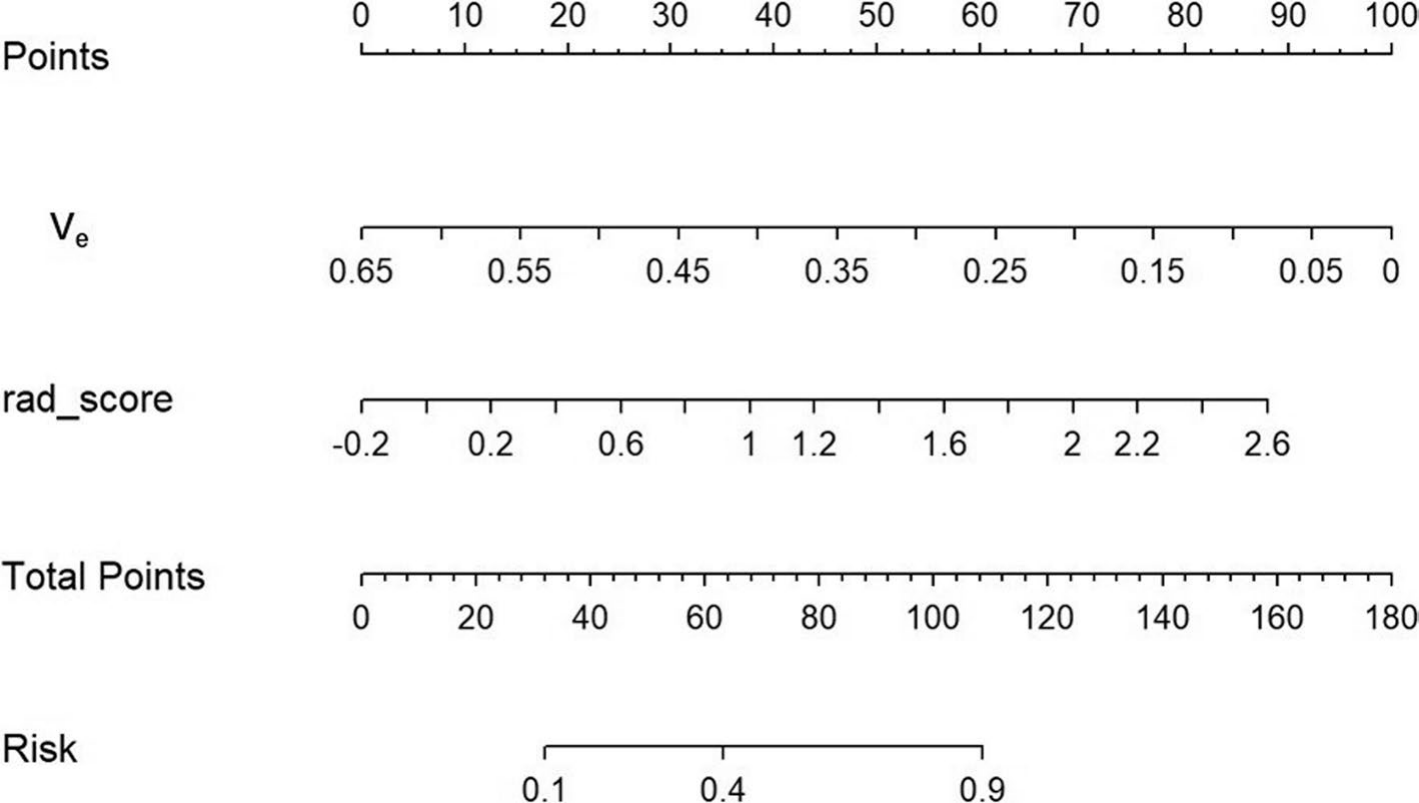
Developed radiomics nomogram generated by combining the features acquired from LASSO and DCE-MRI. The distribution of predictors and the total points are superimposed on the nomogram scales. The density plots show the distribution of continuous variables, such as radiomics signature and total points. The patient and treatment-related characteristics were included in the radiomics nomogram (Sex: 0, female; 1, male; P: TRG 1–5; T: T staging)

A nomogram was generated from the multimodal logistic regression model derived from DCE-MRI, clinical characteristics and radscore. DCE-MRI and radscore were identified as independent factors of the combined model that the VIF of DCE-MRI and radscore is 5.619, 2.835, 7.905 (Fig. 4). The VIF of the formulate of nomogram as follow:

$${\varvec{Y}} = 1/\left( {1 + {\varvec{e}}^{{ - \left( {{\mathbf{2}}{\mathbf{.067}} + {\mathbf{1}}{\mathbf{.805}} \times {\varvec{\beta}}1 + - {\mathbf{8}}{\mathbf{.843}} \times {\varvec{\beta}}2} \right)}} } \right)$$

NOTE: ${\varvec{\beta}}1$** = Rad-score,** ${\varvec{\beta}}2$** = preVemean**.

**Fig. 5:**
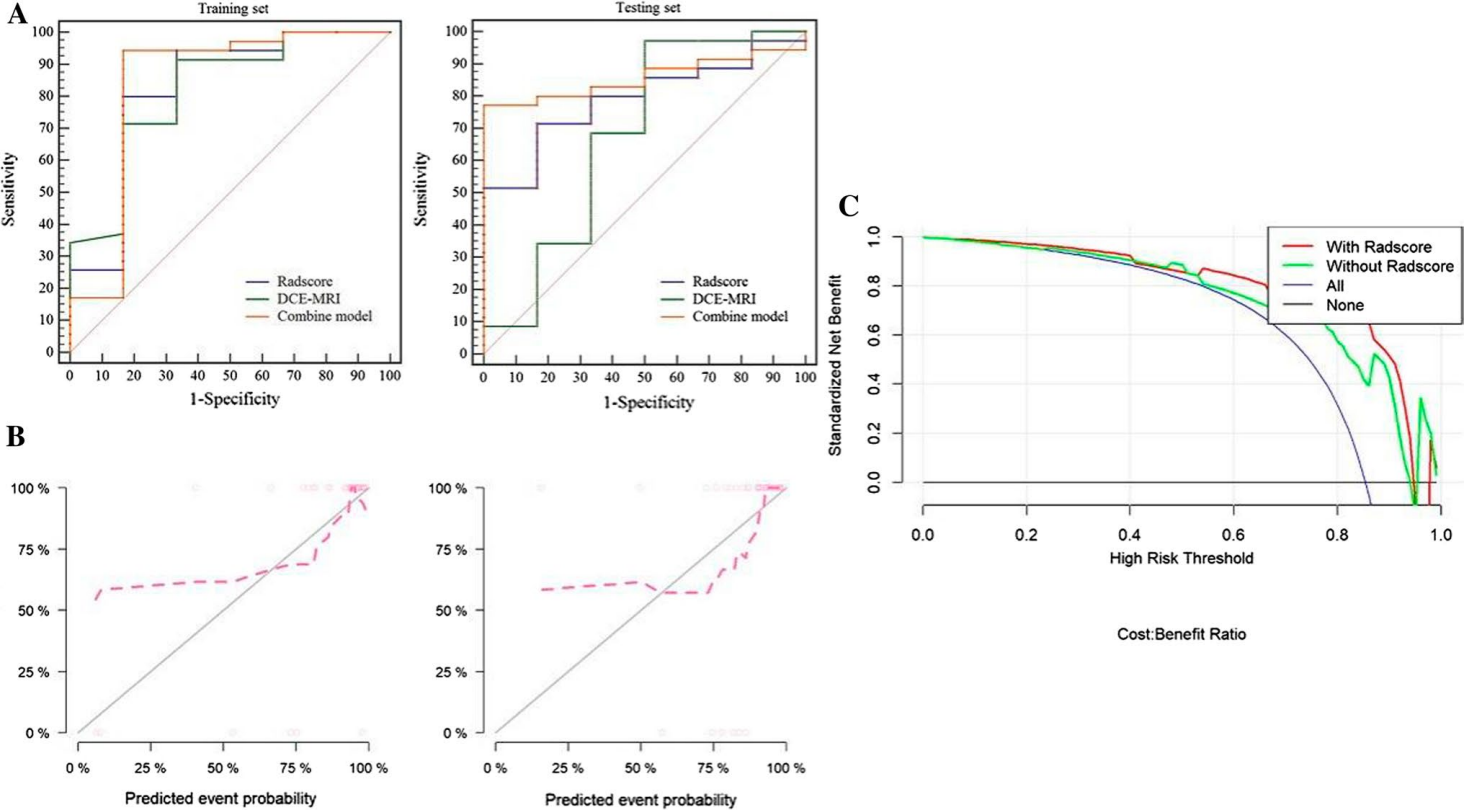
**A** ROC analysis to discriminate responders from non-responders for radscore, clinical model and nomogram. ROC curves for nomogram had the highest area under the ROC curves in both the training set and the test set. **B** calibration curve of nomogram in the training set and the test set. **C** net benefit curves for nomogram compared with models of DCE-MRI andradscore. Y axis means clinical benefit, X axis means the risk of prediction response. None means that none of clinical decision had been taken. All means that random project had been taken. The clinical benefit of nomogram was the highest among nomogram, radscore and clinical model when the clinical risk lower than 0.92

The results showed that the combined radiomics and DCE-MRI nomogram showed the best discrimination ability (AUC 0.84; 95% CI 0.57–1.00 in the training set, AUC 0.86; 95% CI 0.74–0.97 in the test set) that the value of cutoff is 0.5. The radiomics nomogram alone (Rad-score) was able to discriminate responders from non-responders (AUC 0.82; 95% CI 0.59–1.00 in the training set, AUC 0.79; 95% CI 0.63–0.95 in the test set), and DCE-MRI nomogram was able to discriminate responders from non-responders (AUC 0.82; 95% CI 0.61–1.00 in the training set, AUC 0.68; 95% CI 0.36–0.99 in the test set) (Fig. 5A). The AUC of combined model was the highest among the AUCs of Rad-score and DCE-MRI models, and the differences between combined model and Rad-score or DCE-MRI were not significant (*P* = 0.7195 vs 0.7158). The C-index of nomogram in training group was 0.782 and 0.755 in test group. The Hosmer–Lemeshow test in the combined model showed no significant difference in the goodness-of-fit for the Training set and Testing set (*P* = 0.729).

### Clinical usefulness of the radiomics nomogram

As shown in Fig. 5, the clinical impact of the combined DCE-MRI and radiomics nomogram to predict response was observed with maximum utility occurring at 0.95, and DCA could not be analyzed when combining clinical characteristics. For the majority of risk thresholds, the combined DCE-MRI and radiomics model showed the highest net benefit compared with DCE-MRI and radiomics models (Fig. 5). The combine model that combined radiomics and DCE-MRI showed the highest positive and positive predictive value in both training set (0.942, 0.833, respectively) and test set (0.771, 1.000, respectively) (Table 2).

**Table 2:** Predictive ability of different models

	Radscore		DCE-MRI		Combine model	
	Training set	Testing group	Training set	Testing group	Training set	Testing group
AUC	0.824	0.790	0.817	0.676	0.838	0.857
Accuracy	0.805	0.732	0.878	0.854	0.927	0.805
Youden	0.633	0.548	0.581	0.471	0.776	0.771
95% CI						
Lower	0.673	0.635	0.665	0.512	0.690	0.712
Upper	0.925	0.902	0.920	0.814	0.934	0.947
Sensitivity	0.833	0.833	0.941	0.871	0.971	1.000
Specificity	0.800	0.714	0.571	0.500	0.714	0.429
PPV	0.417	0.333	0.914	0.971	0.942	0.771
NPV	0.966	0.962	0.667	0.962	0.833	1.000

## Discussion

A combined radiomics and DCE-MRI nomogram was developed and validated for the pre-nCT prediction of response in patients with EC, which performed well in discriminating responders from non-responders with an AUC of 0.84 in the training set, and similar discrimination with internal validation (AUC = 0.86). The discrimination abilities in the training and test set were comparable, which implied that the nomogram was reliable in quantifying an individual’s risk in non-responders. The combine of radiomics showed the highest PPV in test set (0.971), and the model that combined radiomics and DCE-MRI showed the highest NPV in test set (1.000), however, the specificity of the combined model for test set is the lowest (42.9%). To our knowledge, this is the first MRI radiomics manuscript for predicting response in EC.

The result demonstrated that radiomics nomogram has the potential to decode intratumor heterogeneity on a macroscopic scale noninvasively and quantitatively [17, 18], and genetic alterations or instability can lead to different response to nCT in EC patients [19]. Further radiogenomic analysis is required to validate this hypothesis.

This study showed that the combination of three most significant radiomics features, including MinIntensity, LowGreyLevelRunEmphasis, ClusterShade on pre-nCT MRI, significantly correlated with response and were able to predict response to nCT in EC patients. The minimum intensity value is a basic metric that is commonly used to determine the degree of tumor heterogeneity. Cluster analysis or clustering is the task of grouping a set of objects in which objects in the same group (cluster) are more similar (in some sense or another) to each other than to those in other groups (clusters), which also correlated with the degree of tumor heterogeneity. The grey level run-length matrix (RLM) is defined as the numbers of runs with pixels of gray level i and run length j for a given direction θ, which was correlated with the degree of tumor malignant proliferation. The above three radiomics features proved that tumor heterogeneity and angiogenesis were correlated with response to nCT in EC patients.

Zhang et al. reported that change in the CT value after nCT can predict therapeutic efficacy in EC patients [20]. T2-weighted imaging and Diffusion-weighted imaging were used to assess the residual tumor after nCRT for EC with high sensitivity and low specificity [21, 22]. Intravoxel Incoherent Motion MRI proved useful in early assessment of the response of esophageal squamous cell carcinoma to chemoradiotherapy [23]. PDG-PET response to induction chemotherapy could be a useful imaging biomarker to identify patients with esophageal adenocarcinoma who could benefit from subsequent esophagectomy after chemoradiotherapy [24]. Radiomics had been analyzed for predicting response to neoadjuvant therapy in rectal cancer and cervical cancer patients [9, 10]. However, to date, little is known about the role of DCE-MRI radiomics in assessing response in patients with EC.

In our study, DCE-MRI was performed by a prototype Radial volumetric interpolated breath-hold examination (VIBE) with the time resolution of 2.4 s for most phases, which is a new DCE and provides more phases than conventional sequences. Theoretically, Radial VIBE could acquire more information and with higher time resolution than conventional DCE. Heethuis et al. reported that changes in tumor area-under-the-concentration time curve throughout treatment were promising for predicting histopathologic response to nCRT for EC [4]. Lei et al. reported that K_trans_ prior to chemoradiotherapy, and K_trans_ and Kep at 3 weeks post-treatment are sensitive prediction parameters that are generated using conventional DCE-MRI [25]. Interesting, in the current study, 17 MRI-based texture features on pre-nCT MRI show significance in predicting response of nCT EC.

The present study also has several limitations. Firstly, the data of both training and validation were acquired from the single institution, and further multicenter validation would be our next work. Secondly, the sample size is small, especially for TRG 1 and 2 (35 non-responders/6 responders), which may lead to a certain degree of bias in the results. Because all patients received only 2 cycles of standard nCT protocol followed by surgical resection, which may lead to the majority cases to be non-responders, and this nCT protocol may not have been adequate for pCR. In this study, response rate was 14.6%, which is similar to the report of pathological (20.5%) response rate [26]. However, no significant difference was seen in 3- and 5-year progression-free survival or 3- and 5-year overall survival. The addition of radiotherapy to neoadjuvant chemotherapy results in higher R0 resection rate and pCR rate, without significantly impacting survival [27]. Finally, tumor segmentation especially for small lesion could be challenging. However, 2 readers carefully performed tumor segmentation and discrepancy was resolved by consensus.

## Conclusions

To conclude, we constructed a combined radiomics and DCE-MRI nomogram which is able to reliably discriminate tumor response to nCT, and the radiomics features are useful for nCT response prediction in EC patients. It may provide a convenient tool for clinicians to estimate individuals’ risk of non-response to nCT and to guide treatment personalization for those patients.

## Figures and Tables

**Fig. 6:**
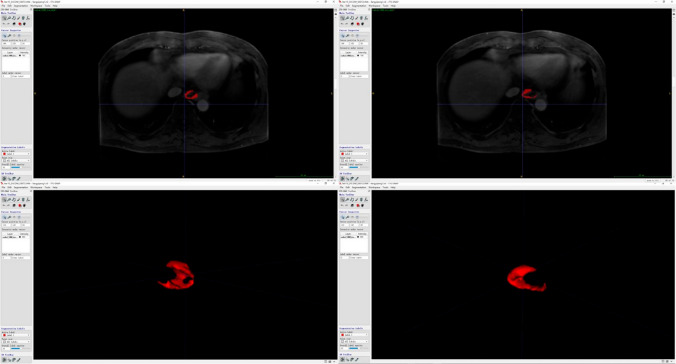
Tumor Segmentation, and 3D tumors of pre-nCT were segmented

## Data Availability

All data generated and analyzed during this study are included in this published article (and its supplementary information files).

## Appendix 1

See Fig. 6, Table 3

**Table 3:** ICC coefficients for all radiomics features

Radiomics features	Coefficients
LongRunEmphasis	0.999378089664866
GreyLevelNonuniformity	0.999361834251752
RunLengthNonuniformity	0.999347482088306
LowGreyLevelRunEmphasis	0.999280953911486
HighGreyLevelRunEmphasis	0.999269180347304
ShortRunLowGreyLevelEmphasis	0.999215212939853
ShortRunHighGreyLevelEmphasis	0.99919967758075
LongRunLowGreyLevelEmphasis	0.999161124400232
LongRunHighGreyLevelEmphasis	0.999137599266105

.

## Abbreviations

TRG: Tumor regression grade
DCA: Decision curve analysis
EC: Esophageal cancer
pCR: Pathologic complete response
DCE: Dynamic contrast enhanced
EGD: Esophagogastroduodenoscopy
AIF: Arterial input function
ROIs: 3D regions of interests
ICC: Inter-class correlation coefficient
VIF: Variance inflation factor
AUC: Area under curve
RLM: Run-length matrix
nCT: Neoadjuvant chemotherapy
